# Hepatic lipocalin 2 promotes liver fibrosis and portal hypertension

**DOI:** 10.1038/s41598-020-72172-7

**Published:** 2020-09-23

**Authors:** Jiegen Chen, Josepmaria Argemi, Gemma Odena, Ming-Jiang Xu, Yan Cai, Veronica Massey, Austin Parrish, Rajanikanth Vadigepalli, Jose Altamirano, Joaquin Cabezas, Pere Gines, Juan Caballeria, Natasha Snider, Pau Sancho-Bru, Shizuo Akira, Ivan Rusyn, Bin Gao, Ramon Bataller

**Affiliations:** 1 Departments of Medicine and Nutrition, University of North Carolina at Chapel Hill, Chapel Hill, NC 27599 USA; 2 Division of Gastroenterology, Hepatology and Nutrition, Pittsburgh Liver Research Center, University of Pittsburgh Medical Center, Pittsburgh, PA 15213 USA; 3 Laboratory of Liver Diseases, National Institute on Alcohol Abuse and Alcoholism (NIAAA), National Institutes of Health, Bethesda, DM 20892 USA; 4 Department of Pathology, Anatomy and Cell Biology, Daniel Baugh Institute for Functional Genomics and Computational Biology, Thomas Jefferson University, Philadelphia, PA 19107 USA; 5 Hepatology-Internal Medicine Department, Hospital Quironsalud Barcelona, Barcelona, Spain; 6 Gastroenterology and Hepatology Department, Research Institute Valdecilla (IDIVAL), University Hospital Marques de Valdecilla, Santander, Spain; 7 Hospital Clinic, Institut D’Investigacions Biomediques August Pi I Sunyer (IDIBAPS), CIBER de Enfermedades Hepáticas Y Digestivas (CIBERehd), Barcelona, Catalonia Spain; 8 Department of Cell Biology and Physiology, School of Medicine, University of North Carolina at Chapel Hill, Chapel Hill, NC 27599 USA; 9 Laboratory of Host Defense, Immunology Frontier Research Center, Osaka University, Suita, Osaka Japan; 10 Department of Veterinary Integrative Biosciences, Texas A&M University, College Station, TX 77843 USA

**Keywords:** Liver diseases, Alcoholic liver disease, Liver fibrosis

## Abstract

Advanced fibrosis and portal hypertension influence short-term mortality. Lipocalin 2 (LCN2) regulates infection response and increases in liver injury. We explored the role of intrahepatic LCN2 in human alcoholic hepatitis (AH) with advanced fibrosis and portal hypertension and in experimental mouse fibrosis. We found hepatic *LCN2* expression and serum LCN2 level markedly increased and correlated with disease severity and portal hypertension in patients with AH. In control human livers, LCN2 expressed exclusively in mononuclear cells, while its expression was markedly induced in AH livers, not only in mononuclear cells but also notably in hepatocytes. *Lcn2*^−/−^ mice were protected from liver fibrosis caused by either ethanol or CCl_4_ exposure. Microarray analysis revealed downregulation of matrisome, cell cycle and immune related gene sets in *Lcn2*^−/−^ mice exposed to CCl_4_, along with decrease in *Timp1* and *Edn1* expression. Hepatic expression of *COL1A1*, *TIMP1* and key *EDN1* system components were elevated in AH patients and correlated with hepatic *LCN2* expressio*n.* In vitro*,* recombinant LCN2 induced *COL1A1* expression. Overexpression of *LCN2* increased HIF1A that in turn mediated *EDN1* upregulation. LCN2 contributes to liver fibrosis and portal hypertension in AH and could represent a new therapeutic target.

## Introduction

Excessive alcohol consumption is responsible for 3.8% of global mortality^1^. Among alcohol-induced organ damage, alcohol-related liver disease (ALD) is a major cause of morbidity and mortality^2^. ALD progresses from fatty liver to hepatic inflammation, progressive fibrosis and hepatocellular carcinoma. Moreover, patients with heavy alcohol use and underlying ALD can present episode(s) of alcoholic hepatitis (AH), which is the most severe form of ALD. AH is characterized by an abrupt development of jaundice, liver failure and portal hypertension^3^. Mortality in AH patients remains very high, and targeted therapies beyond corticosteroids are urgently needed. We previously found that advanced fibrosis and portal hypertension determine the prognosis of AH^4,5^. Uncovering the cellular and molecular mechanisms underlying fibrosis in AH could favor the development of novel therapies.

LCN2, also known as neutrophil gelatinase-associated lipocalin (NGAL), is a secreted 25-kDa glycoprotein belonging to lipocalin superfamily^6,7^ first identified as a protein stored in specific granules of human neutrophils^8^. LCN2 has multiple functions in regulation of innate immunity^9^, cell proliferation^10^, apoptosis^11^, metabolism^12,13^ and tumor metastasis^14^. LCN2 is a pro-inflammatory cytokine and a useful biomarker of acute kidney injury^10,15^. Hepatic LCN2 is markedly increased in experimental liver injury and its increase is mediated by pro-inflammatory cytokines^16^. The role of LCN2 in liver inflammation is inconsistent. Mice lackcing *Lcn2* show more liver injury after CCl_4_, ConA and LPS exposure^17^. However, LCN2 deficiency protects against alcohol and diet-induced liver injury^18,19^. The relevance of LCN2 in liver fibrogenesis and portal hypertension is unknown. Activation of hepatic stellate cell is a landmark in fibrosis because these cells turn into the primary source of extracellular matrix in liver upon injury. In vitro, recombinant LCN2 is reported to induce type 1 collagen protein expression in human fibroblasts in a dose-dependent fashion^20^. In cultured mouse collecting duct cells, silencing of LCN2 receptor represses transforming growth factor beta 1 (TGFβ1) signaling and α-smooth muscle actin expression^21^. These findings suggest that LCN2 could act as an extracellular stimulus to modulate trans-differentiation of quiescent retinol-storing cells into fibrogenic myofibroblasts. We hypothesized that hepatic LCN2 was involved in activation of HSCs in AH patients.

Here, we describe the association of LCN2 hepatic and circulatory levels with liver fibrosis, portal hypertension and disease severity in patients with AH. Using ethanol-fed and CCl_4_-induced liver fibrosis in *Lcn2*-deficient mice, we demonstrate that LCN2 plays a key role in liver fibrogenesis and portal hypertension. Moreover, we provide evidence that this effect is mediated by hypoxia-induced factor 1 (HIF1A)/EDN1 axis.

## Results

### LCN2 gene expression and serum levels in AH: correlation with disease severity, liver fibrosis and portal hypertension

**Figure 1:**
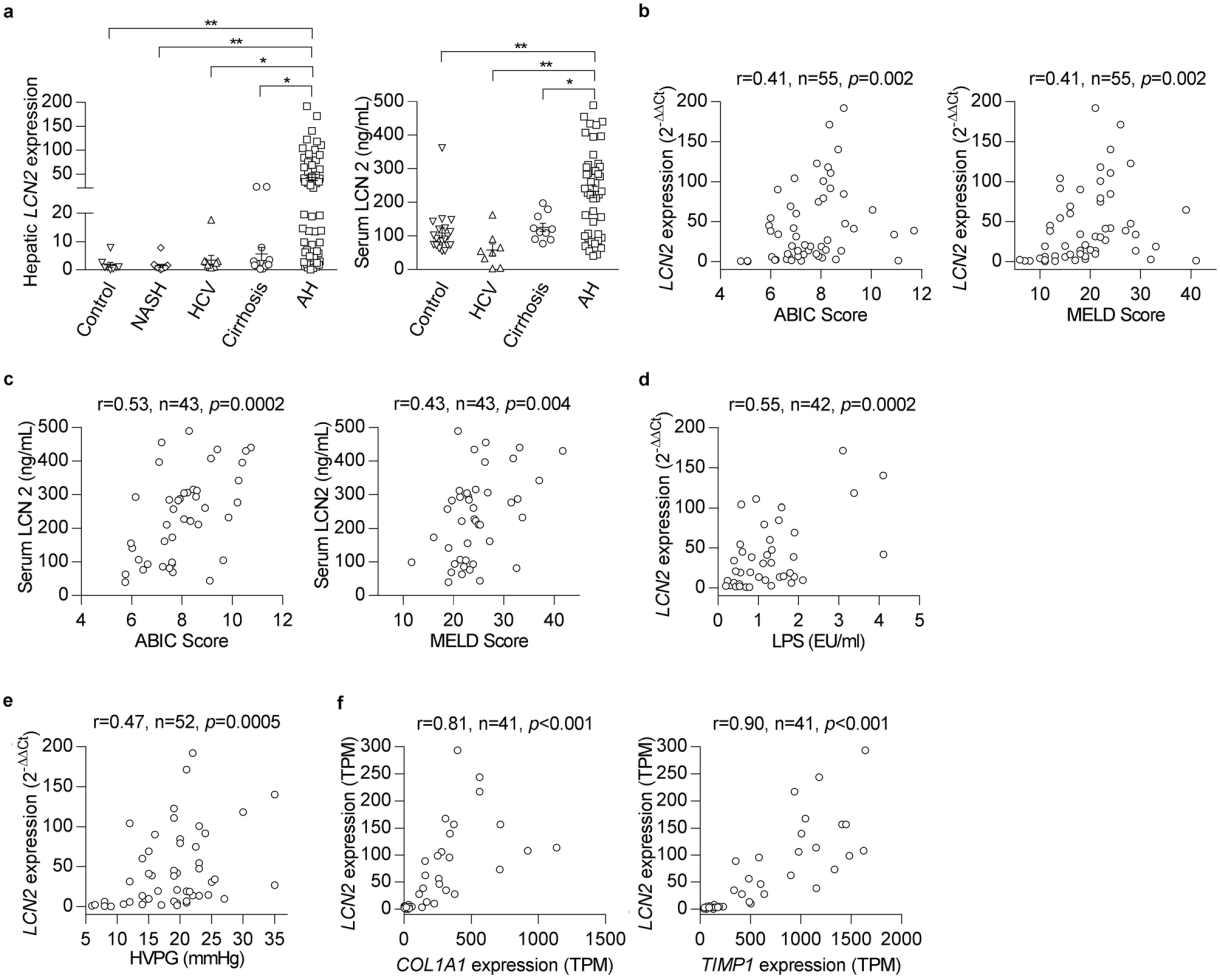
*LCN2* gene expression and serum levels of LCN2 increase in patients with AH and correlate with the disease severity, portal hypertension and pro-fibrogenic gene expression. (**a**) Hepatic *LCN2* mRNA expression was measured by qPCR in normal controls (n = 8), NASH (n = 14), HCV (n = 10), cirrhosis (n = 13) and patients with AH (n = 55). Serum LCN2 level was determined by ELISA in normal controls (n = 20), HCV (n = 8), cirrhosis (n = 10) and patients with AH (n = 45). (**b**) Hepatic *LCN2* gene expression correlated with ABIC and MELD scores. (**c**) Serum level of LCN2 correlated with ABIC and MELD scores. (**d**) Hepatic *LCN2* gene expression correlated with the level of circulating LPS. (**e**) Hepatic *LCN2* gene expression correlated with the degree of portal hypertension. (**f**) Hepatic *LCN2* mRNA expression correlated with *COL1A1* and *TIMP1* gene expression. *p < 0.05; **p < 0.01 by one-way ANOVA.

Analysis of microarray data from patients with AH^22^ showed that hepatic *LCN2* gene expression is one of the most up-regulated genes in the whole transcriptome (Supplementary Figure 1a). Confirmatory real-time qPCR showed a dramatic up-regulation of *LCN2* in AH patients, which was not observed in other liver diseases including nonalcoholic steatohepatitis (NASH), HCV-induced chronic hepatitis and HCV-induced compensated cirrhosis. In patients with AH, serum LCN2 levels correlated with the hepatic *LCN2* mRNA expression (Fig. 1a). The results suggest that hepatic *LCN2* gene expression is an important source of circulating LCN2 in patients with AH.

To assess the clinical relevance of LCN2, the association of *LCN2* gene expression with parameters indicative of disease prognosis was evaluated. We found that hepatic *LCN2* gene expression positively correlated with the main prognostic scores in patients with AH, including the Age/Bilirubin/International normalized ratio/Creatinine (ABIC) score (r = 0.41, *p* = 0.002) and the Model for End-stage Liver Disease (MELD) score (r = 0.41, *p* = 0.002) (Fig. 1b). Moreover, serum level of LCN2 positively correlated with AH disease severity, as assessed by the ABIC score (r = 0.53, *p* = 0.0002) and MELD score (r = 0.43, *p* = 0.004) (Fig. 1c).

It is well-established that Gram-negative bacterium-derived LPS is a major driver of AH^23^. We found that hepatic *LCN2* gene expression in patients with AH closely correlated with circulating LPS levels (r = 0.55, *p* = 0.0002) (Fig. 1d). Furthermore, in vivo, both hepatic *Lcn2* mRNA expression and serum LCN2 increased after LPS injection in mice (Supplementary Figure 1b). LPS exposure induced LCN2 expression around sixfold in precision-cut rat liver slices (Supplementary Figure 1c). In contrast, we found that gene expression levels of hepatic LCN2 receptors (i.e. *SLC22A7* and *LRP2*) were slightly increased in AH patients (Supplementary Figure 1d and e).

Importantly, hepatic *LCN2* mRNA expression in patients with AH closely correlated with the degree of portal hypertension (r = 0.47, *p* = 0.0005) (Fig. 1e), a major pathophysiological event. The analysis of RNA-sequencing data from a similar cohort of AH patients revealed that hepatic *LCN2* gene expression was highly associated with two extracellular matrix genes: *COL1A1* (r = 0.81, *p* < 0.001) and *TIMP1* (r = 0.90, *p* < 0.001) (Fig. 1f). These results strongly suggest that LCN2 may play a pathogenic role in liver fibrosis and portal hypertension in patients with AH.

### Hepatocyte LCN2 expression is highly induced in human AH but not in mouse ALD model

**Figure 2:**
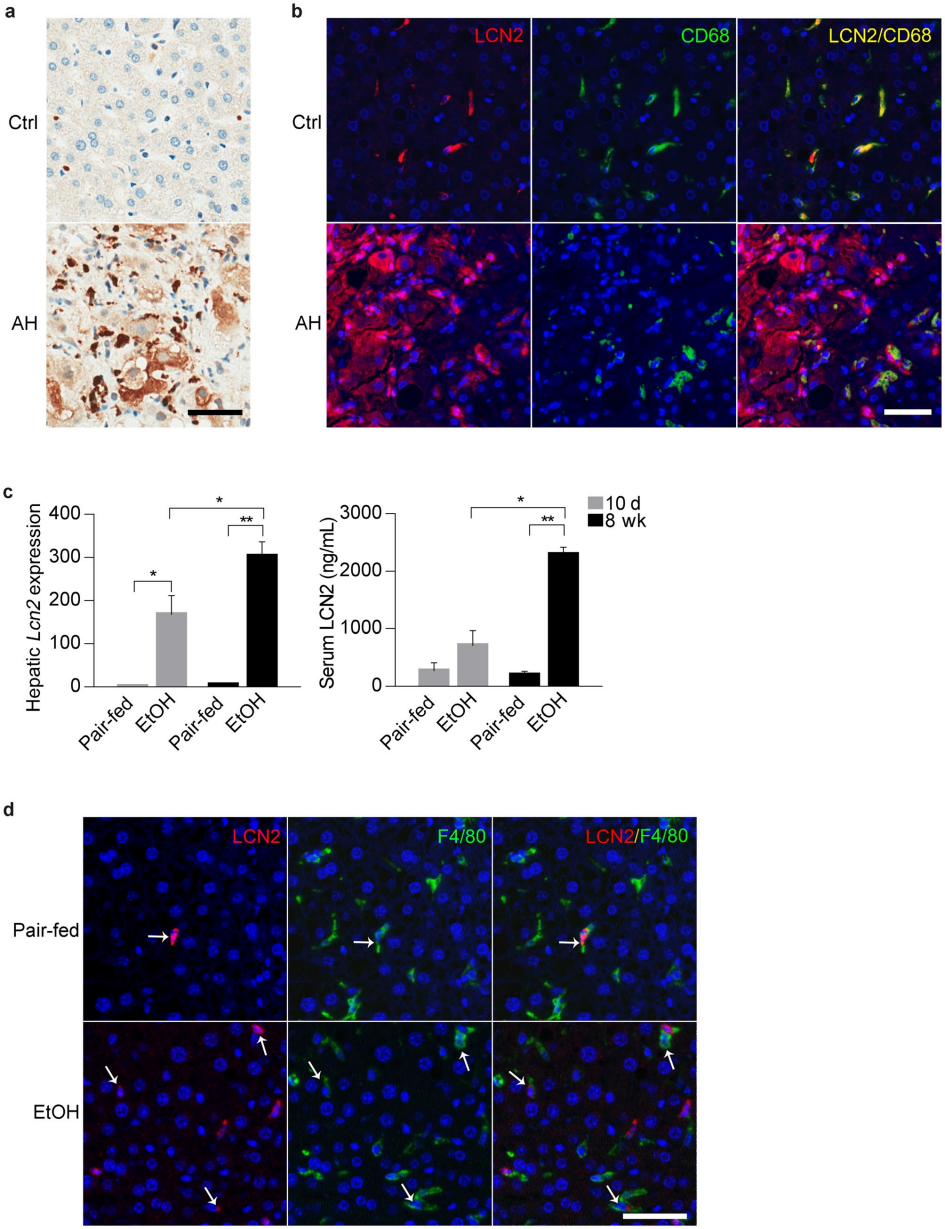
LCN2 expression in human AH and mouse ALD. (**a**) Representative IHC images of LCN2 expression in the liver from normal control (Ctrl, n = 9) and patients with AH (AH, n = 6). (**b**) Representative IF images of co-staining of LCN2 and CD68 expression in the biopsies from control (Ctrl, n = 6) and patient with AH (AH, n = 4). Hoechst 33,258 (blue) used to label nuclei, TSA-Cy5 visualized LCN2 (red) and TSA-Cy3 visualized CD68 (green). Overlay of LCN2 and CD68 showed dual expression (yellow). (**c**) Hepatic *Lcn2* expression and serum levels of LCN2 in mice after 10-day (grey) or 8-week (black) ethanol exposure plus binge (n = 4–5). (**d**) Representative IF images of dual staining showed co-localization of LCN2 and F4/80 expression in the liver from pair-fed and ethanol-fed mice (n = 4–5). Hoechst 33,258 (blue) used to label nuclei, TSA-Cy5 visualized LCN2 (red) and TSA-Cy3 visualized F4/80 (green). Overlay of LCN2 and F4/80 showed dual expression (blue/green). *p < 0.05; **p < 0.01 by one-way ANOVA. Scale bar: 50 μm.

To determine the cell source of LCN2 in AH, we performed IHC in the liver sections from normal controls and patients with AH. In normal livers, LCN2 exclusively expressed in mononuclear cells while its expression was markedly increased in AH livers, not only in inflammatory cells but also notably in hepatocytes (Fig. 2a). Macrophages have been described as LCN2 secreting cells in mice^9^ and Kupffer cells are resident macrophages in human liver. Concordantly, immunofluorescence staining showed that LCN2 expression co-localized with CD68, a human macrophage cell marker (Fig. 2b). In line with these findings, cultured hepatocytes *LCN2* mRNA expression induced by LPS dose-dependently. In macrophages *LCN2* gene expression increased markedly with LPS treatment (Supplementary Figure 2a and b).

We next assessed LCN2 expression in mice subjected to sub-acute and chronic experimental ALD. For this purpose, mice were fed an ethanol diet plus one binge of ethanol for 10-day or 8-week, as previously described^24^. Upon ethanol treatment, hepatic *Lcn2* mRNA expression significantly increased in both models. Serum LCN2 level was higher in the 8-week ethanol-fed mice (Fig. 2c). LCN2-positive macrophages (Fig. 2d) and neutrophils (Supplementary Figure 2c) were increased in 8-week ethanol-fed mice, but the increase of LCN2 expression not shown in hepatocytes. Take together these results suggest a species discrepancy and that ethanol exposure increases hepatic LCN2 expression only from inflammatory cells in mice. However, in human AH, LCN2 not only from inflammatory cells but also highly induced in hepatocytes.

### LCN2 ablation attenuates CCl_4_-induced fibrosis by mediating ECM deposition and G-protein-coupled receptor (GPCR) signaling

**Figure 3:**
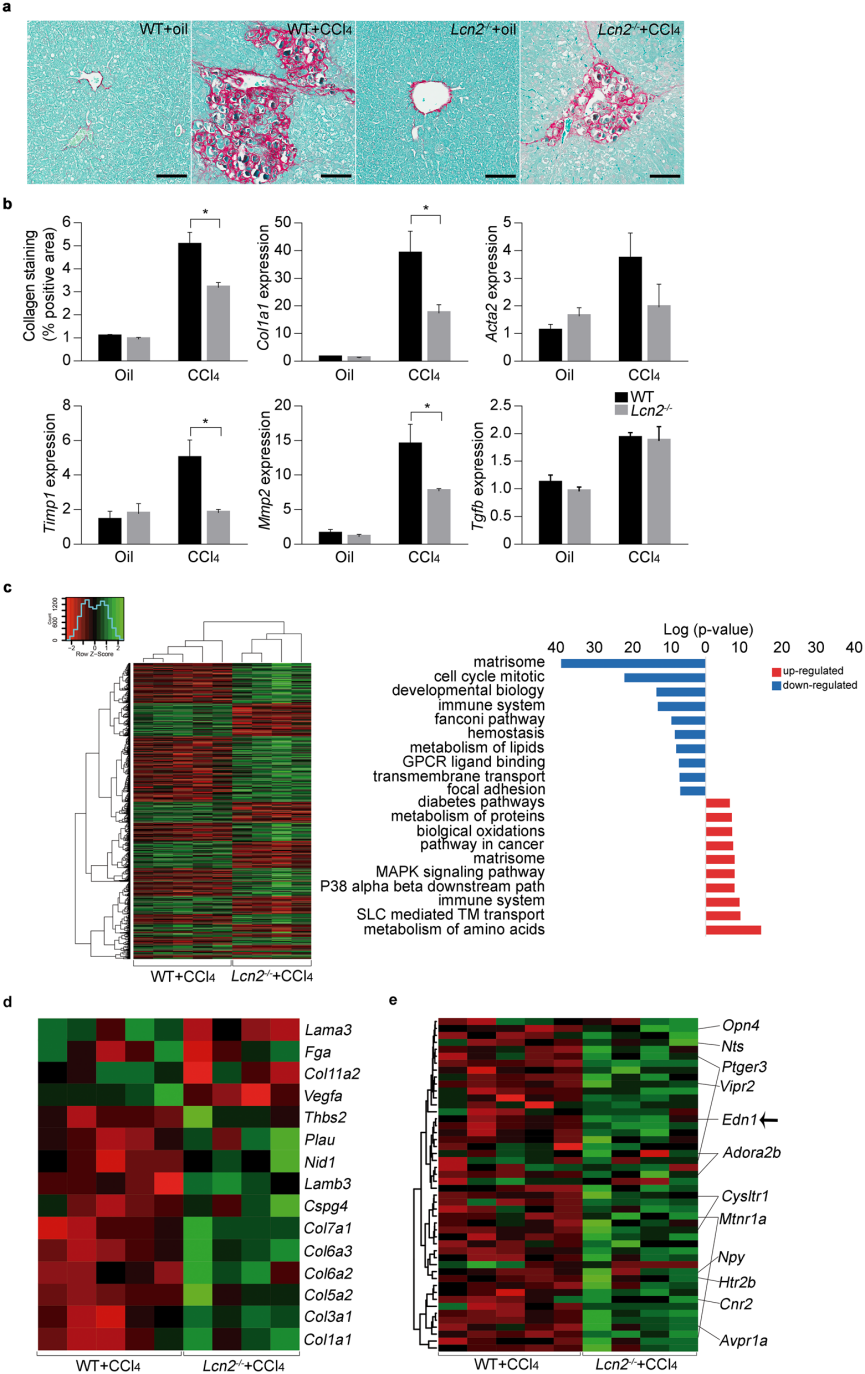
*Lcn2*^−/−^ mice are protected from chronic CCl_4_ exposure induced liver fibrosis. (**a**) BALB/c WT and *Lcn2*^−/−^ mice exposed to CCl_4_ for 4 weeks and representative images of collagen fibers stained with Sirius red (n = 11–19). (**b**) The surface area stained with Sirius red was quantitated using digital image analysis. *Col1a1, Acta2*, *Timp1*, *Mmp2* and *Tgfb1* gene expression was quantified by qPCR (n = 14–20). (**c**) Gene set enrichment analysis for WT and *Lcn2*^−/−^ mice treated with CCl_4_ and functional pathways identified (n = 4–5). (**d**) Heatmap of genes of Integrin pathway that were differentially regulated in WT and *Lcn2*^−/−^ mice treated with CCl_4_ (n = 4–5). (**e**) Heatmap of genes of GPCR binding pathway differentially regulated in WT and *Lcn2*^−/−^ mice treated with CCl_4_ (n = 4–5). *p < 0.05 by one-way ANOVA. Scale bar: 100 μm.

To explore the potential role of LCN2 in the development of liver fibrosis, WT and *Lcn2*^−/−^ mice were exposed to chronic ethanol for 8 weeks plus binge administration. Even in the absence of overt fibrosis in this model (Supplementary Figure 3a), we found that *Timp1* expression, an important pro-fibrogenic effector, was significantly decreased in ethanol-fed *Lcn2*^−/−^ mice compared with ethanol-fed WT mice, pointing to this metalloprotease inhibitor as an early mechanism in alcoholic liver fibrosis (Supplementary Figure 3b). To further study the role of LCN2 in liver fibrosis, we used CCl_4_ for 4 weeks to induce advanced fibrosis in WT and *Lcn2*^-/–^ mice. Notably, *Lcn2*^–/–^ mice developed less fibrosis accumulation compared with WT littermates (Fig. 3a). The induction of expression of *Col1a1* and the genes involved in extracellular matrix turnover, such as alpha-smooth muscle actin (*Acta2*), *Timp1* and *Mmp2* were abrogated in mice lacking *Lcn2* (Fig. 3b).

To dissect the molecular mechanisms that mediating LCN2-induced fibrogenic effects in the injured liver, we performed transcriptome profiling analysis in the liver of *Lcn2*^–/–^ and WT mice treated with CCl_4_. Absence of *Lcn2* gene attenuated the transcriptome changes induced by CCl_4_. The number of significantly up-regulated genes (1,346 in WT *vs* 1,067 in *Lcn2*^−/−^) and downregulated genes (991 in WT *vs* 562 in *Lcn2*^−/−^) was significantly decreased in mice lacking *Lcn2* gene. Gene set enrichment analysis of the differentially expressed genes (GSEA) revealed, among downregulated functions, an enrichment of gene sets related to matrisome, cell cycle, development and immune response (Fig. 3c). A detailed analysis of these gene sets showed the lack of upregulation of multiple collagen genes and genes encoding ECM adhesion glycoproteins in CCl_4_-treated *Lcn2*^−/−^ mice (Fig. 3d). Another pathway affected by *Lcn2* knockout was G-protein-coupled receptors (GPCR) signaling (Fig. 3e). Defects in GPCR regulation have severe consequences affecting GPCR-stimulated vascular responses^25^. These results suggest that CCl_4_-mediated upregulation of ECM and endothelin system genes are dependent on *Lcn2* gene expression and LCN2 is a key pathogenic regulator in mouse CCl_4_-induced liver fibrosis.

### Endothelin system gene expression is increased in mouse ALD and human AH

**Figure 4:**
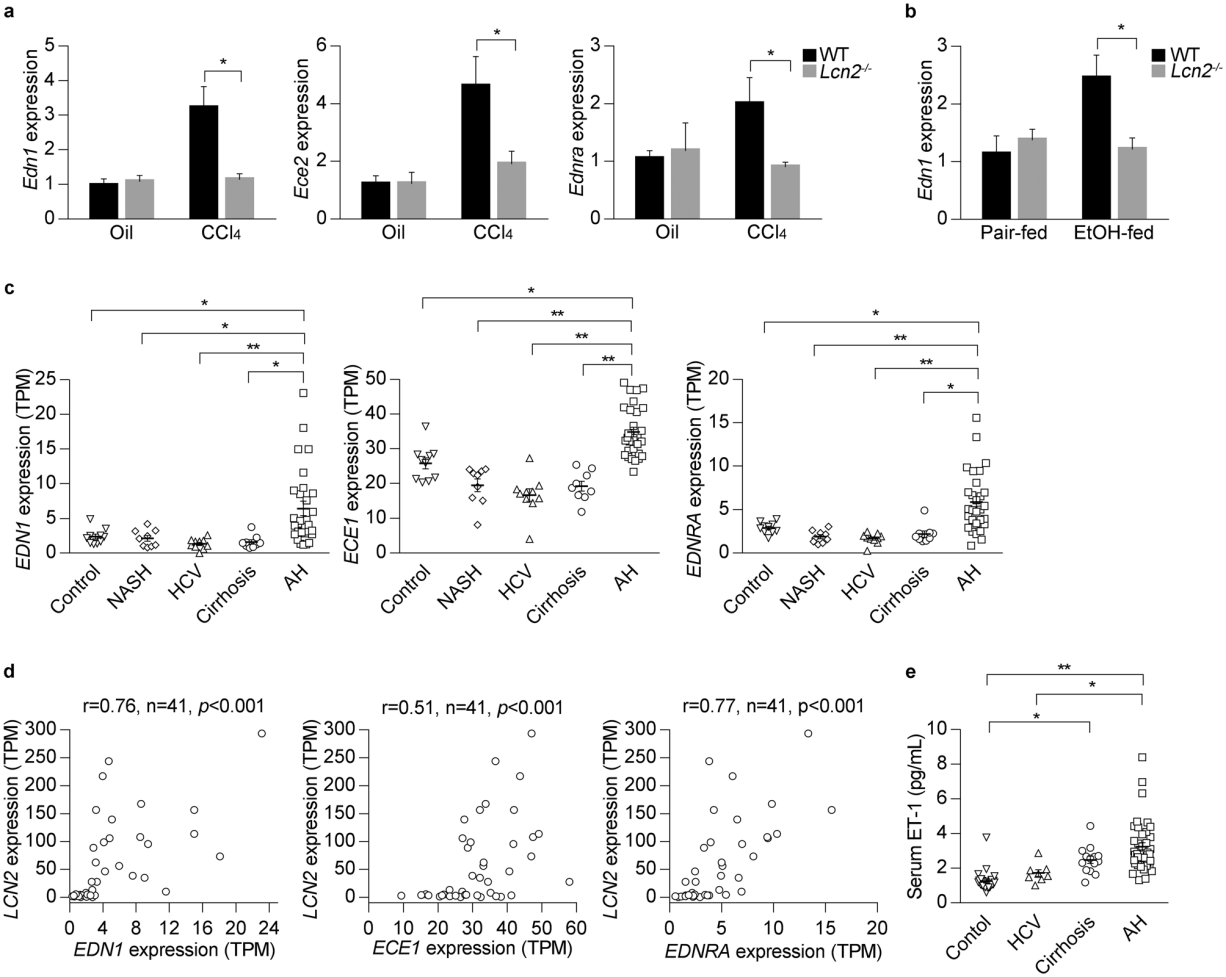
Expression of endothelin system genes increases in mouse experimental model and human AH. (**a**) Hepatic endothelin system gene expression was confirmed by qPCR from WT and *Lcn2*^−/−^ mice exposed to CCl_4_ (n = 4–6). (**b**) Hepatic endothelin system gene expression from WT and *Lcn2*^−/−^ mice after 8-week ethanol plus binge exposure. (n = 4 for each group). (**c**) Human liver biopsies for RNA-sequencing analysis of *EDN1*, *ECE1* and *EDNRA* expression included multiple etiologies of liver tissues representing control liver (n = 10), NASH (n = 9), HCV (n = 10), compensated cirrhosis (n = 9) and AH (n = 29). (**d**) Correlation of hepatic *LCN2* and *EDN1*, *ECE1* and *EDNRA* expression in AH patients. (**e**) Circulating ET1 concentration was quantified by ELISA in serum of patients with multiple etiologies of liver disease. *p < 0.05; **p < 0.01 by one-way ANOVA.

We hypothesized that LCN2 could affect splanchnic vascular tone through regulation of vasoconstrictors and vasodilators related to GPCR signaling. ET1, a potent vasoconstrictor, is upregulated in human cirrhosis^26,27^. ET1 activity is regulated by endothelin converting enzymes 1 and 2 (*ECE1* and *ECE2*) and its action is mediated by two different GPCRs: endothelin receptor Type A (*EDNRA*) and Type B (*EDNRB*). Strikingly, mouse *Edn1* gene expression was markedly elevated in WT compared with *Lcn2*^−/−^ mice upon CCl_4_ treatment (Fig. 4a). We then explored the expression of the endothelin system gene in 8-week plus binge ethanol-fed mice. In this model, chronic ethanol exposure was sufficient to induce *Edn1* expression in WT but not in *Lcn2*^−/−^ mice (Fig. 4b). Furthermore, we studied the expression of endothelin system genes in the liver of patients with AH. RNA-sequencing data showed that the expression of *EDN1*, *ECE1* and *EDNRA* was specifically increased in AH livers compared with normal livers and other diseased livers (Fig. 4c). Interestingly, the levels of these transcripts were highly correlated with hepatic *LCN2* expression (Fig. 4d). Circulating ET1 concentration was higher in AH patients than in normal controls and HCV patients (Fig. 4e). These results indicate that the endothelin system gene expression is activated in mouse and human after alcohol exposure and is tightly correlated with *LCN2* gene expression in the liver.

### LCN2-HIF1Α axis regulates endothelin expression in human hepatic stellate cells (HSCs)

**Figure 5:**
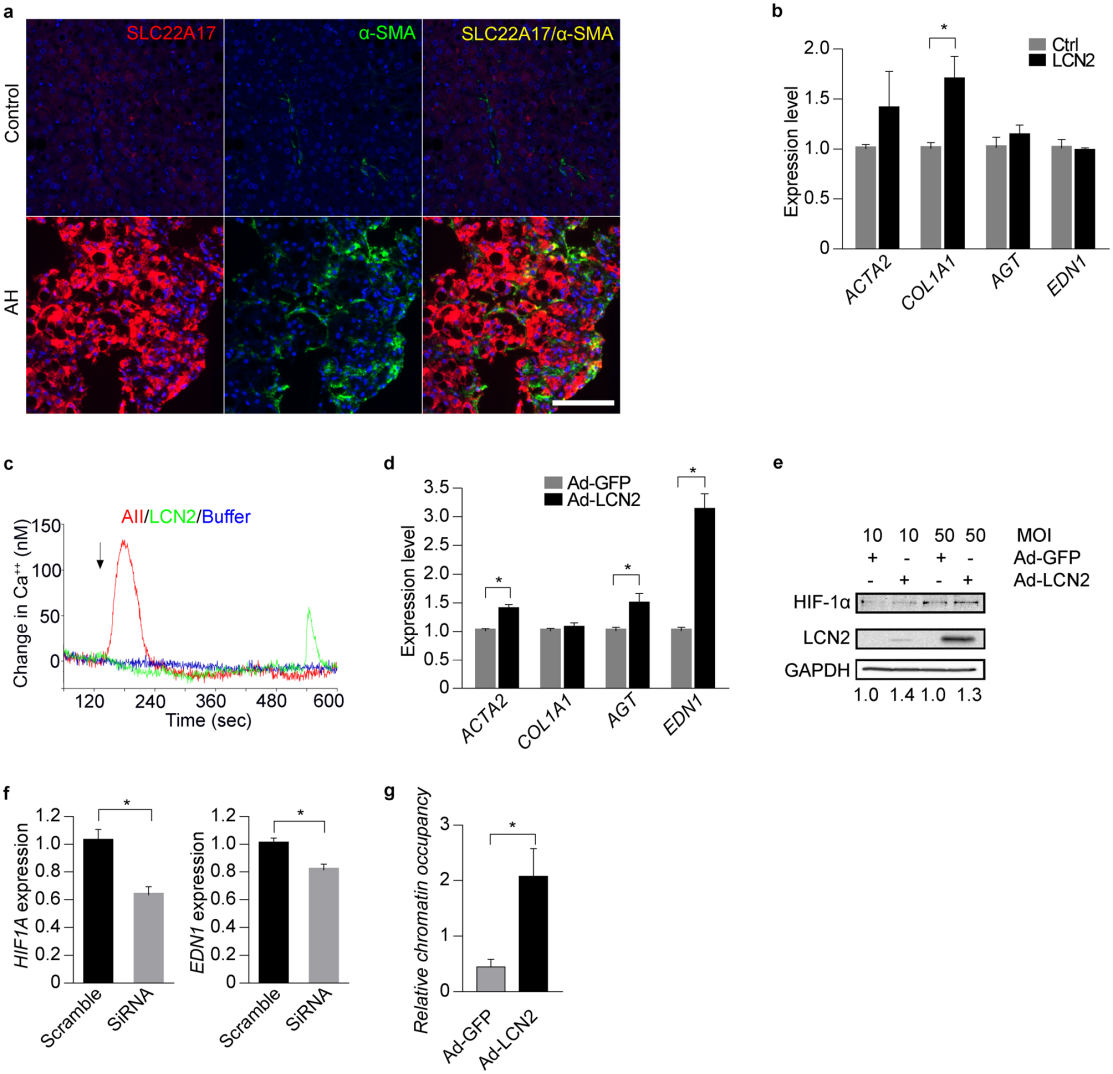
LCN2-HIF1A pathway regulates *EDN1* expression in human HSCs. (**a**) Representative IF images of co-localization LCN2 receptor (*SLC22A17*) and α-SMA in AH livers. Hoechst 33,258 (blue) was used to label nuclei, TSA-Cy5 visualized *SLC22A17* (red) and TSA-Cy3 visualized α-SMA (green). Overlay of *SLC22A17* and α-SMA showed co-expression (yellow). (**b**) LX-2 cells were exposed to 50 µM human recombinant LCN2. *ACTA2*, *COL1A1*, *AGT* and *EDN1* gene expression was measured by qPCR (n = 3). (**c**) Intracellular free calcium concentration of LX-2 cells was detected using Fura-2 fluorescence. (**d**) Human primary HSCs transduced with adenovirus-mediated overexpression of GFP or LCN2. *ACTA2*, *COL1A1*, *AGT* and *EDN1* gene expression were measured by qPCR (n = 3). (**e**) Human primary HSCs transduced with adenovirus-mediated overexpression of GFP or LCN2. HIF1A expression was detected by Western Blot (n = 3) and the original blots are presented in Supplementary Figure 5. (**f**) *HIF1A* siRNA was transfected into human primary HSCs and *EDN1* gene expression measured by qPCR (n = 3). (**g**) ChIP-qPCR was used to detect binding affinity of HIF1A to the *EDN1* gene promoter in human primary HSCs (n = 3). *p < 0.05 by two-tailed Student’s *t* test. Scale bar: 100 μm.

We next explored whether LCN2 could induce the synthesis of ET1 in the human liver cells. In patients with AH, immunofluorescence staining showed that both hepatocytes and HSCs expressed SLC22A17 (LCN2 receptor) (Fig. 5a). In vitro, we used adenovirus-mediated overexpression of LCN2 in human primary hepatocytes (HPHs) and HepG2 cells. RNA-sequencing data showed that the expression profiles were not significantly altered in both transduced cells (Supplementary Figure 4a). We then investigated the effect of LCN2 on cultured human primary HSCs. Treatment of HSCs with recombinant LCN2 increased the expression level of gene related to HSC activation (Fig. 5b) and increased intracellular free calcium concentration (61.7 ± 6.0 nM), indicating the presence of active receptors (Fig. 5c and Supplementary Table 2). Furthermore, adenovirus-mediated overexpression of LCN2 in the HSCs increased expression of *END1* along with HSCs activation marker *ACTA2* and other vasoconstrictor factors like angiotensinogen (*AGT*) (Fig. 5d).

We finally explored the molecular mechanism involved in LCN2-induced END1 up-regulation in the HSCs. In breast cancer cell LCN2 can induce HIF1A expression, which is an important transcriptional factor mediated *EDN1* gene expression in endothelial cells^28,29^. Therefore, we used human LCN2 over-expression adenovirus to infect the HSCs and Western blot results showed that LCN2-overexpression induced *HIF1A* expression (Fig. 5e). Transfecting specific *HIF1A* siRNA into the HSCs to knockdown HIF1A expression (Supplementary Figure 4b), we found the decreased *EDN1* mRNA expression in the cells (Fig. 5f). Consistently, ChIP-qPCR data showed the increased binding activity of HIF1A to the promoter region of *EDN1* gene in HSCs-overexpressing *LCN2* (Fig. 5g). Taken together, these results indicate LCN2 induces the increase of *EDN1* expression mediated by HIF1A in human HSCs.

## Discussion

We previously demonstrated that the presence of advanced fibrosis/cirrhosis in patients with AH confers a bad prognosis^4^. Complications in AH are due to liver failure and the development of severe portal hypertension. It is known that severe fibrosis is a key determinant of portal hypertension. In fact, previous reports have shown that the degree of portal hypertension, as assessed by HVPG, correlates with short-term survival^30^. Most investigations of the pathogenesis of AH have focused on intrahepatic inflammation and hepatocellular failure. The current study was undertaken to identify druggable molecular drivers of fibrogenesis and portal hypertension in AH. Through liver transcriptome analysis, we identified LCN2 as one of the most up-regulated genes in AH patients^22^. Recent studies showing that liver-derived LCN2 is a prognostic factor in a series of patients with acute-on-chronic liver failure, half of them being AH patients^31^. We provide evidence that LCN2 is massively overexpressed in the liver from patients with AH and its expression correlates with the degree of fibrosis and portal hypertension. Because some pro-inflammatory mediators also play a role in fibrosis development and the progression of portal hypertension^32^, we analyzed the potential pro-fibrogenic role of LCN2 in human AH and experimental animal model. Animal models of ethanol-induced liver injury do not completely develop many of the key features of advanced ALD, including the development of massive fibrosis and liver failure^3^. To overcome this limitation, we assessed the functional role of LCN2 in human samples and in mice subjected to a well characterized model of liver fibrosis. The findings that hepatic LCN2 expression closely correlated with disease severity (i.e. ABIC and MELD scores), the degree of portal hypertension (i.e. levels of HVPG) and the expression of key fibrogenic genes (i.e. *COL1A1*, *TIMP1*) highly suggest a pathogenic role for LCN2 in AH. Mouse lacking LCN2 showed an attenuated fibrogenic response to liver injury. These results reveal a novel pathogenic function for LCN2 in chronic liver diseases with potential therapeutic implications.

Our studies clearly demonstrate that the cell sources of LCN2 in human advanced ALD are not only from inflammatory cells but also from hepatocytes. This notion is important since reprogrammed hepatocytes are able to produce pro-inflammatory and fibrogenic mediators such as IL-8 and now LCN2^33^. The fact that fibrosis in AH is characterized by a pericellular pattern highly suggest that hepatocytes play a major role in fibrosis development^34^. Maneuvers aimed at preventing the expression and secretion of fibrogenic mediators by hepatocytes may have beneficial effects. Interestingly, we also show that LPS mediates LCN2 overexpression in hepatocytes. This finding confirms previous data from our group showing that LPS is an important mediator in AH and supports the notion that targeting LPS could have beneficial effects in AH^35,36^. It is notable that *LCN2* gene is expressed in early stages of rat primary HSC activation. In these primary cells, protein levels of LCN2 were highest when HSCs were fully activated^37^. These findings strongly suggest that LCN2 could be a fibrogenic mediator in the chronically damaged liver.

We partially uncovered the mechanisms of LCN2-induced fibrosis and portal hypertension in AH. In microarray analysis in mice lacking *Lcn2* gene with attenuated fibrogenic response, we identified ET1 as a potential mediator of LCN2 in AH. Previous studies have shown that ET1 is a fibrogenic factor that promotes portal hypertension in the damaged liver^38,39^. In fact, plasma ET1 levels are elevated in patients with cirrhosis, and there is a positive association with poor prognosis and increased portal pressure^26,27^. The sources of ET1 in the damaged liver include endothelial cells and HSCs^40–43^. Because HSCs are liver-specific pericytes that play a key role linking fibrosis and portal hypertension, we demonstrated that LCN2 promotes *EDN1* synthesis in HSCs during liver fibrogenesis. While wild type mice with advanced fibrosis showed a marked increase in *Edn1* hepatic expression, *Lcn2* deficient mice did not show such upregulation. Notably, LCN2 also mediated the expression of key genes for ET1 generation and biological actions, including *Ece2* or *Ednra*. These results suggest a key role of LCN2 in ET1 regulation in the fibrotic liver. Interestingly, chronic ethanol plus binge model showed an increase in the expression of *Edn1*. In this model, LCN2 was again necessary for *Edn1* upregulation. Our results indicate that alcohol exposure can stimulate the LCN2-mediated expression of *Edn1*. In this study, due to technique difficulties in measuring portal pressure in CCl_4_-treated mice, we could not confirm the effect of LCN2 on portal hypertension in experimental models.

Previous studies in alcohol-induced steatohepatitis in mice unfolded the role of HIF1A as a key inducer of liver injury^44^. Ethanol and acetaldehyde are reported to induce HIF1A -mediated expression of *Edn1* in endothelial cells (28). Our study showed LCN2-overexpression in HSC induced HIF1A expression. Importantly, decreasing *HIF1A* expression levels in HSC resulted in reduced *EDN1* expression. The binding activity of HIF1A to the promoter region of *EDN1* increased in HSCs overexpressing LCN2. ET1 could be one of HIF1A targets responsible for its downstream actions in fibrosis and angiogenesis^45^. Our results add new evidence to the role of HIF1A in liver injury.

In summary, our results suggest that LCN2 massive hepatic expression in patients with AH could favor fibrosis and portal hypertension, two of the main predictors of patients’ morbidity and mortality. The LCN2-HIF1A-ET1 axis is a potential new mechanism of hepatocyte-HSC communication in chronic ethanol-induced liver damage. Further studies should evaluate if drugs interfering with LCN2 synthesis and/or biological actions have beneficial effects in patients with this severe clinical condition.

## Materials and methods

### Human subjects

All human biospecimens were obtained from the National Institute on Alcohol Abuse and Alcoholism (NIAAA)-funded InTeam Consortium Human Biorepository Core (University of Pittsburgh, PA). All human studies conformed to the ethical guidelines of the Declaration of Helsinki and were approved by the Institutional Review Board of the University of Pittsburgh. Patients with biopsy-proven AH as well as normal controls were included as described in detail previously^46^. Informed consent was obtained from all human participants. The clinical characteristics of the patients included in Supplementary Table 1.

### Experimental animal studies

All the experiments reported in this study were adherent to the Animal Research: Reporting of In Vivo Experiments (ARRIVE) guidelines. The protocols for animal housing, treatment and euthanasia were approved by the Institutional Animal Care and Use Committee of the University of North Carolina at Chapel Hill, and the NIAAA Animal Care and Use Committee and the Institutional Animal Care and Use Committee of Texas A&M University.

*LPS injection.* Eight-week-old male BALB/c mice were injected intravenously by a single dose of LPS at 10 mg/kg body weight. Mice were sacrificed at 6 h after injection^35^.

*Subacute and chronic ethanol exposure plus binge. Lcn2*^*–/–*^ mice were back-crossed to a C57BL/6N background for at least nine generations. Mice were maintained on a normal chow diet in the NIAAA animal facility. Eight- to ten-week-old male *LCN2*^–/–^ and C57BL/6N mice were initially fed a controlled Lieber-DeCarli diet ad libitum for 5 days to acclimatize them to a liquid diet. Subsequently the ethanol-fed groups were allowed free access for 10 days or 8 weeks to an ethanol diet containing 5% (vol/vol) ethanol. Binge feeding was a single dose of ethanol (5 g/kg body weight) via gavage in the early morning. Mice were sacrificed 9 h later^47,48^.

*Chronic CCl4 treatment.* BALB/c wild type and *Lcn2*^−/−^ mice were generously provided by Dr. Shizuo Akira from Osaka University, Japan. *Lcn2*^−/−^ mice were generated on a 129/Ola X C57BL/6 (B6.129) background as described previously^9^ and backcrossed to BALB/c mice for at least nine generations^49^. Six- to eight-week-old male BALB/c WT or *Lcn2*^−/−^ mice were subjected to CCl4 which diluted into corn oil (1:5) intraperitoneal injection for 4 weeks at dose of 0.5 ml/kg body weight twice a week. Mice were sacrificed 48 h after last dose of CCl4 administration.

### Microarray studies

Human liver microarray studies were performed as previously described^35^. For mouse microarray, liver tissue was collected from BALB/c WT and *Lcn2*^−/−^ mice treated with CCl4 or corn oil for 4 weeks. Total RNA was isolated and the integrity and purity were determined. Complementary DNA were labelled with Fluoresce Cy3 and hybridized to SurePrint G3 mouse gene expression chip slide (Agilent, G4852B). The arrays were scanned and analyzed for a digital probe count comparison. Quantile normalization, multidimensional scaling, principal component analysis and hierarchical clustering were performed using base R functions. Differential expression analysis were performed by means of *limma* package^50^. In order to analyze RNA expression differences between WT and *Lcn2*^−/−^ mice treated with CCl4, Benjamini–Hochberg was used as an adjustment method for multiple comparisons. For gene set enrichment analysis (GSEA) top 2000 up and downregulated annotated probes were used. Probe annotation was made by using Agilent probe identification information 028,005-D gene list, version 20,171,030. Heatmaps of top differentially expressed genes (DEG) and of selected lists of genes was performed by using heatmap.2 function of *gplots* package. GSEA analysis included the DEG overlap with Molecular Signatures Database gene sets (Broad Institute, v 6.1), computed by using GSEA on-line web tool. Hypergeometric distribution was calculated to detect most enriched gene sets. We focused our analysis on Canonical Pathway gene sets, from Curated Gene Sets collection^51^.

### RNA sequencing

RNA sequencing was performed using Illumina HiSeq2000 platform (San Diego, CA) in human liver samples as described previously^46^. Total RNA samples were also obtained from transduction with adenovirus vectors containing either human LCN2 or a GFP sequence in human primary hepatocytes and HepG2 cells.

### Cell culture

*Human primary hepatic stellate cells (HSCs*)*.* Human primary HSCs purchased from ScienCell Research Laboratories (Catalog #5300) was isolated and purified from human liver. The cells were seeded and cultured according to manufacturer’s instruction. Transduction with adenovirus vectors containing either human LCN2 (Ad-LCN2, MOI = 10, 50) or a GFP sequence (Ad-GFP) (Signagen, SL112614) were performed on HSCs for 48 h. To knockdown *HIF1A* gene expression*,* transfection with 50 nM either scrambled or *HIF1A* small interfering RNA (Thermo Fisher Scientific, AM16708A) for 48 h were performed with jetPRIME transfection reagent.

### ChIP-qPCR analysis

chromatin DNA was prepared using EpiTect ChIP Kit (Qiagen, 334471). Briefly, cells were transduced with GFP- or LCN2- expressing adenovirus and cultured for 72 h. Then cells were treated with 1% formaldehyde at 37 °C to cross-link proteins to DNA. The samples were sonicated to fragments (Branso Digital Sonifer, model 102c). Anti-HIF1A (H1alpha67) monoclonal antibody (Thermo Fisher Scientific, MA116511) was used to immunoprecipitate binding fragments. Control IgG and polymerase II monoclonal antibody (Qiagen, 334481) was used as a negative and positive control respectively. The precipitated chromatin was then treated with proteinase K to reverse cross-links. DNA was subjected to real-time PCR with primers for human *EDN1* gene promoter (Qiagen, GPH1011088 (-) 01A) or positive control (Qiagen, GPH110001C( +)01A) with SYBR Green ROX Mastermix (Qiagen, 33052).

### Immunohistochemistry (IHC) and immunofluorescence (IF) staining

Human liver specimens from patients with AH and healthy liver fragments, and mouse liver sections were used to study LCN2 expression. IHC was performed using the Bond Fully-automated Slide Staining System. Slides were dewaxed in Bond Dewax solution and hydrated in Bond Wash solution. Heat-induced antigen retrieval was performed for 30 min at 100 °C in Bond-Epitope Retrieval solution 1 pH 6.0 (Leica Biosystems, AR9961). Antigen retrieval was followed by 5 min Bond peroxide blocking step. After pretreatment, slides were incubated with primary antibody anti-LCN2. Chromogenic detection of antibodies was performed using the Bond Polymer Refine Detection System (Leica Biosystems, DS9800). Positive and negative controls were included for each run.

Dual IF staining was performed in the same system except antigen retrieval for F4/80 in Bond enzyme1 (Leica Biosystems, AR9551) for 5 min at 37 °C. The primary antibodies information is shown in the Supplemental Table 3. Stained slides were counterstained nwith Hoechst 33,258 and mounted with ProLong Diamond Antifade Mountant.

### Serum LCN2 and ET1 determination

Serum was obtained through peripheral blood extraction from patients with AH (n = 26), HCV (n = 24), NASH (n = 23), compensated alcoholic cirrhosis (n = 17) and healthy controls (n = 30). The samples were analyzed following the instructions provided from the Quantikine ELISA human LCN2 immunoassay kit (R&D Systems, DLCN20) and ET1 Immunoassay kit (R&D Systems, DET100).

### Statistical analyses

Results of quantitative variables are expressed as mean ± SEM unless otherwise specified. Statistical analysis was performed using GraphPad Prism 7 software. Comparisons between groups were performed using the Student’s *t* test or one-way ANOVA. Correlations between variables were evaluated using Spearman’s rho or Pearson’s r, when appropriate. A *p* value less than 0.05 was considered statistically significant.

## Supplementary information

Supplementary Information

## Data Availability

The microarray data has been deposited in NCBI's Gene Expression Omnibus (GEO) under Accession Number GSE130123. The RNA sequencing data has been deposited in NCBI GEO under Accession Number GSE130128.
