# Supplementary material for: Hepatic lipocalin 2 promotes liver fibrosis and portal hypertension
Source: Sci Rep. 2020 Sep 23;10:15558. doi: 10.1038/s41598-020-72172-7 (PMC7512007; doi:10.1038/s41598-020-72172-7)

Additional information

Hepatic Lipocalin 2 Promotes Liver Fibrosis and Portal Hypertension

Jiegen Chen[1#], Josepmaria Argemi[2#], Gemma Odena[1], Ming-Jiang Xu[3], Yan Cai[3], Veronica Massey[1], Austin Parrish[4], Rajanikanth Vadigepalli[4], Jose Altamirano[5], Joaquin Cabezas[6], Pere Gines[7], Juan Caballeria[7], Natasha Snider[8], Pau Sancho-Bru[7], Shizuo Akira[9], Ivan Rusyn[10], Bin Gao[3] and Ramon Bataller[1,2 *]

[#] Both authors contributed equally to the work.

[1] University of North Carolina at Chapel Hill, Departments of Medicine and Nutrition, Chapel Hill, NC 27599, USA

[2] University of Pittsburgh Medical Center, Division of Gastroenterology, Hepatology and Nutrition, Pittsburgh Liver Research Center, Pittsburgh, PA 15213, USA

[3] National Institutes of Health, Laboratory of Liver Diseases, National Institute on Alcohol Abuse and Alcoholism (NIAAA), National Institutes of Health, Bethesda, DM 20892, USA

[4] Thomas Jefferson University, Daniel Baugh Institute for Functional Genomics and Computational Biology, Department of Pathology, Anatomy and Cell Biology, Philadelphia, PA 19107, USA

[5] Hospital Quironsalud Barcelona, Hepatology-Internal Medicine Department, Barcelona, Spain

[6] University Hospital Marques de Valdecilla, Gastroenterology and Hepatology Department, Research Institute Valdecilla (IDIVAL), Santander, Spain

[7] Hospital Clinic, Institut d'Investigacions Biomediques August Pi i Sunyer (IDIBAPS), CIBER de Enfermedades Hepáticas y Digestivas (CIBERehd), Barcelona, Catalonia, Spain

[8] University of North Carolina at Chapel Hill, Department of Cell Biology and Physiology, School of Medicine, Chapel Hill, NC 27599, USA

[9] Osaka University, Laboratory of Host Defense, Immunology Frontier Research Center, Suita, Osaka, Japan

[10] Texas A&M University, Department of Veterinary Integrative Biosciences, College Station, TX 77843, USA

Corresponding author: Ramon Bataller, MD, PhD, Division of Gastroenterology, Hepatology and Nutrition, Pittsburgh Liver Research Center, University of Pittsburgh Medical Center, Pittsburgh, PA 15213, USA. Tel: (412) 647-4932. E-mail: bataller@pitt.edu

Methods

RNA isolation and quantitative RT-PCR

Total RNA was isolated by using RNeasy mini kit (Qiagen, 74104) for mouse tissues or using Trizol reagent (Thermo Fisher Scientific, 15596-018). Tissues (< 30 mg) were homogenized by Power Gen 125 (Thermo Fisher Scientific). RNA quality and concentration were determined with Nanodrop 2000C spectrophotometer. Complementary DNA was synthesized by using high capacity cDNA reverse transcription kit (Applied Biosystems, 4368814) according to the manufacturer's protocol. Complementary DNA was analyzed by qPCR (StepOnePlus, Applied Biosystems) and 18S was used as an internal control. Each qPCR included a 10 µL reaction mixture per well containing 2µL cDNA, 0.5µL Gene Expression Assay primers and 1X TaqMan Fast Advance Master Mix reagent (Thermo Fisher Scientific, 4444556). Gene expression was calculated using comparative Ct method ($2^{-\Delta\Delta Ct}$).

Precision-cut rat liver slices

250µm high-precision cut slices were obtained from freshly isolated livers using a vibratome instrument VT1000S (Leica Microsystems), as described previously[35]. Liver samples were washed in PBS and soaked and oriented in a 4% agarose solution. Agarose blocks were mounted and fixed using cyanocrilate glue on the vibratome and submerged in HBSS to perform the cutting process. Splicing speed and frequency parameters were used as the optimized method. Slices were released from the agarose and plated above cell culture inserts containing warmed culture medium. Slices were further stabilized in a humidified incubator at 37ºC and 5% CO_2 overnight. After the stabilization period, slices treated with 50 ng/ml LPS for 24 hours.

Immunoblot

Cells were lysed in RIPA buffer (Sigma, R0278) containing a protease inhibitor cocktail (Roche, 5892970001). Proteins were resolved by SDS–PAGE and then transferred onto nitrocellulose membranes. Membranes were incubated at 4°C overnight with the following primary antibodies: HIF1A (Cell Signaling Technology, 3716S), LCN2 (LifeSpan BioSciences, LS-C332404) and GAPDH (Abcam, ab8245). Appropriate secondary antibodies used to detect bound primary antibodies. The membranes were developed using Luminata Crescendo Western HRP substrate (Millipore, WBLUR 0500) on the FluorChem E system (ProteinSimple) and quantified by Image J (the ratio of HIF1A intensity/GAPDH intensity).

Sirius Red staining

For Sirius Red staining, FFPE sections of mouse liver tissue were deparafinized and rehydrated. Slides were incubated in saturated picric acid containing 0.1% Sirius Red and 0.1% Fast Green for 30 min. The slides were scanned on an Aperio ScanScope XT. Images were uploaded to eSlide Manager and visualized with ImageScope 12.3.2 (Leica Biosystems). Whole tissue sections were then analyzed with the Aperio Color Deconvolution v9 algorithm configured as a custom macro for this assay. Sirius Red and Methyl Green were sampled to obtain representative Optical Density values for the RGB color space. These values were used as inputs to mark the appropriate stains. Intensity thresholds were set to distinguish negative, weak Positive, medium positive and strong positive pixels for the Sirius Red only. Data output was presented as percent positive.

Cell cultures

LPS treatment. The murine macrophage cell line RAW246.7 (ATCC, TIB-71) and the hepatocyte cell line SNU449 were cultured and treated with indicated dose of LPS (Sigma-Aldrich, L4391) for 16-18 hours.

LCN2 exposure and cellular free calcium determination. Recombinant human LCN2 50 μM (R&D systems, 1757-LC) was added to LX-2 cells for 16-18 hours. For intracellular Ca^{++} measurement, cells were plated on 25-mm diameter glass and serum starved for 24 hours. Cells were subsequently washed in incubation buffer (121 mM NaCl, 10 mM HEPES, 5 mM $NaHCO_3$, 4.7 mM KCl, 1.2 mM KH_2PO_4, 1.2 mM $MgSO_4$, 1.25 mM $CaCl_2$, 10 mM glucose, and 1.8% bovine serum albumin at pH 7.4). 50μM Fura-2 AM (Thermo Fisher Scientific, F1221) was added and incubated in 37°C for 25 minutes. Then the modified incubation buffer containing 0.01% BSA washed off unincorporated Fura-2. Addition of 10^{-8}M angiotensin II (AII), TCN buffer (50 mM Tris, 10 mM $CaCl_2$, 150 mM NaCl, pH 7.5) or 50 μM recombinant human LCN2, then Fura-2 fluorescence was detected using a CCD camera (Digital Video Camera Co. DVC 1500) after passing through an emission filter. Intracellular Ca^{++} was determined by ratiometric analysis of Fura-2 emission intensities at 340nm and 380nm with InCytIm2 software (Intracellular Imaging). The data was presented as average increase of intracellular Ca^{++} concentration from all responder cells.

Immunofluorescence of HIF1A in human primary HSCs

Human primary HSCs cultured according to manufacturer's protocol (ScienCell Research Laboratories, 5300). To knockdown *HIF1A* expression, transfection with

50nM either scrambled or *HIF1A* small interfering RNA (Thermo Fisher Scientific, AM16708A) for 72 hours were performed with jetPRIME transfection reagent. Cells were washed briefly with PBS and 10% formalin fixed for 10 min. Epitope retrieved 30mins by citrate buffer. Anti-HIF1A monoclonal antibody (BD Transduction Laboratories, 610959) was used to detect HIF1A expression at 1:100 dilution for 5 hours incubation. Detection of bound primary antibodies were accomplished with TSA-Cy5. Nuclear was counterstained with Hoechst 33258.

Supplemental Tables

Table 1. Baseline characteristics of patients with AH.

Characteristics	Median (25-75 interquartile range)
Age (years)	51 (46-59)
Male n (%)	43 (78)
**Laboratory and hemodynamic parameters**	
Hemoglobin (g/dL)	12 (10-14)
Leukocyte count x10^9 /L	8.5 (6.3-14.9)
Platelet count x10^9/L	114 (76-231)
AST (U/L)	117 (67-232)
ALT (U/L)	37 (25-69)
Serum albumin (g/dL)	2.6 (2.3-3.2)
Serum creatinine (mg/dL)	0.88 (0.65-1.16)
Serum bilirubin (mg/dL)	6.1 (2.6-23.4)
HVPG (mmHg)	19 (14-22)
LPS (EU/ml)	1.12 (0.54-1.89)
**Alcoholic hepatitis severity scores at admission**	
MELD score	19 (14-24)
ABIC score	7.3 (6.7-8.4)
**Clinical decompensations during hospitalization**	
Infection n (%)	13 (24)
SIRS (%)	19 (36)
AKI n (%)	19 (35)
**Fibrosis**	
S1: Perisinusoidal or periportal n (%)	2 (3.6)
S2: Perisinusoidal/portal/periportal n (%)	0 (0)
S3: Bridging fibrosis n (%)	6 (10.9)

S4: Cirrhosis n (%)	47 (85.5)
**Mortality at 90 days** n (%)	10 (18)

Table 2. The number of responder cells with intracellular free Ca^{++} increase.

	# of responder cells	# of analyzed cells	Responder cells (%)
Buffer	1	32	3.1
AII	20	42	47.6
LCN2	23	46	50.0

Table 3. The application order of antibodies for IF co-staining

Stain order	Antibody	Vendor	Cat#	Dilution
CD68-LCN2	CD68	Leica Microsystems Inc.	PA0273	RTU
F4/80-LCN2	F4/80	eBioscience	14-4801-82	1:2000
LCN2-Ly6G	LCN2	LifeSpan BioSciences, Inc.	LS-C332404	1:100
SLC22A17-α-SMA	SLC22A17	Sigma-Aldrich,	HPA049718	1:2000
	Ly6G	Abcam	ab25377	1:200
	α-SMA	Abcam	ab5694	1:1000

Supplementary figure legends

Supplementary figure 1. Hepatic *LCN2*, *LRP2* and *SLC22A17* expression in human AH and hepatic *Lcn2* gene expression in mice and rat liver slice.

(a) Comparison of transcriptional levels in the most significantly different expressed genes between patients with AH (n=16) and normal controls (Ctrl, n=7).

(b) Hepatic *Lcn2* mRNA expression (n=4) and serum LCN2 level (n=6) in mice after LPS exposure by tail vein injection.

(c) LCN2 mRNA expression in precision-cut rat liver slices treated with LPS (n=3).

(d) Hepatic *SLC22A17* gene expression was determined by qPCR in normal controls (n=5), NASH (n=9), HCV (n=6), compensated cirrhosis (n=6) and patients with AH (n=31).

(e) Hepatic *LRP2* gene expression was determined by qPCR in normal controls (n=8), NASH (n=7), HCV (n=8), compensated cirrhosis (n=5) and patients with AH (n=33).

ns: not significance, *p< 0.05; by two-tailed Student's *t* test or one-way ANOVA when appropriate.

Supplementary figure 2. *LCN2* gene expression in SNU449 hepatocytes, macrophages and neutrophils.

(a) *LCN2* gene expression in SNU449 hepatocytes treated with LPS (right, n=3).

(b) *Lcn2* gene expression in RAW246.7 macrophage treated with LPS (left, n=4).

(c) Representative images of IF staining showed co-localization of LCN2 and Ly6G expression in the liver from pair-fed and ethanol-fed mice. Hoechst 33258 (blue) used to

label nuclei, TSA-Cy5 visualized LCN2 (red) and TSA-Cy3 visualized Ly6G (green). Overlay of LCN2 and Ly6G showed co-expression (yellow).

*p< 0.05; by one-way ANOVA. Scale bar: 50μm.

Supplementary figure 3. *Lcn2*$^{-/-}$mice are protected from chronic ethanol exposure.

(a) C57BL/6N WT and *Lcn2*$^{-/-}$ mice were fed with ethanol for 8 weeks plus binge. Representative images of collagen fibers were stained with Sirius red (n=4 for each group).

(b) The surface area stained with Sirius red was quantitated using digital image analysis. *Col1a1*, *Acta2, Timp1*, *Mmp2* and *Tgfb1* gene expression was quantified by qPCR.

*p< 0.05 by one-way ANOVA. Scale bar: 100μm.

Supplementary figure 4. The effect of LCN2 on human hepatocytes and knockdown of HIF1A expression in HSCs.

(a) Average counts for each transcript from control (Ad-GFP) and LCN2-transfected cells from human primary hepatocytes (HPH) and HepG2 cells (n=3).

(b) *HIF1A* siRNA was transfected into human primary HSCs. Representative IF images of HIF1A staining. Scale bar: 50μm.

Supplementary figure 5. Original western blots.

(a) HIF1A **(b)** LCN2 **(c**) GAPDH.

Supplementary figure 1

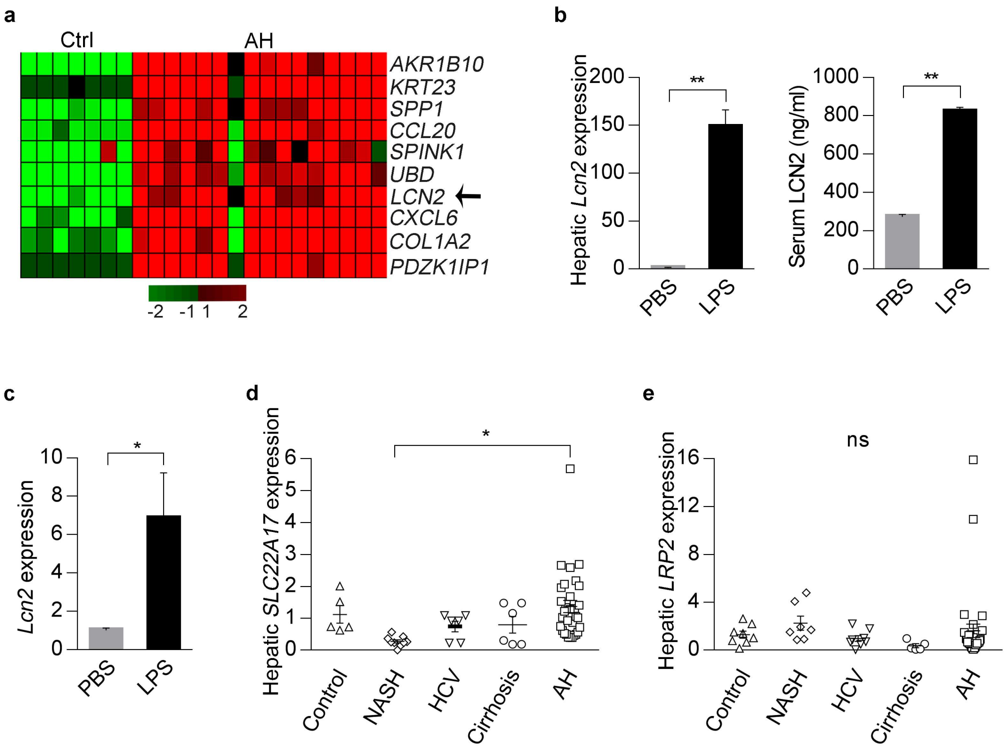

Supplementary figure 2

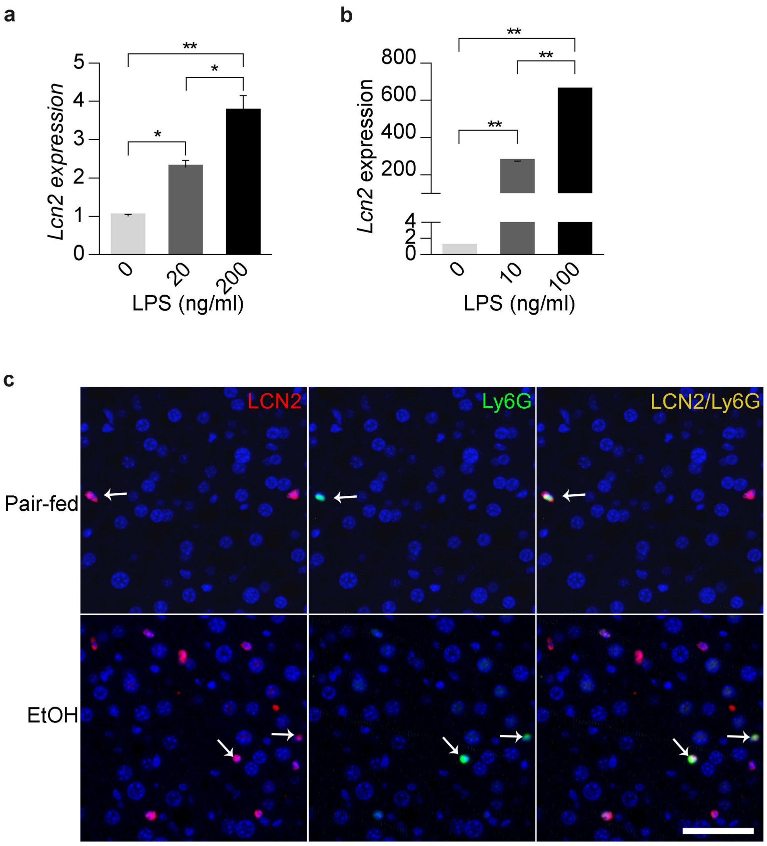

Supplementary figure 3

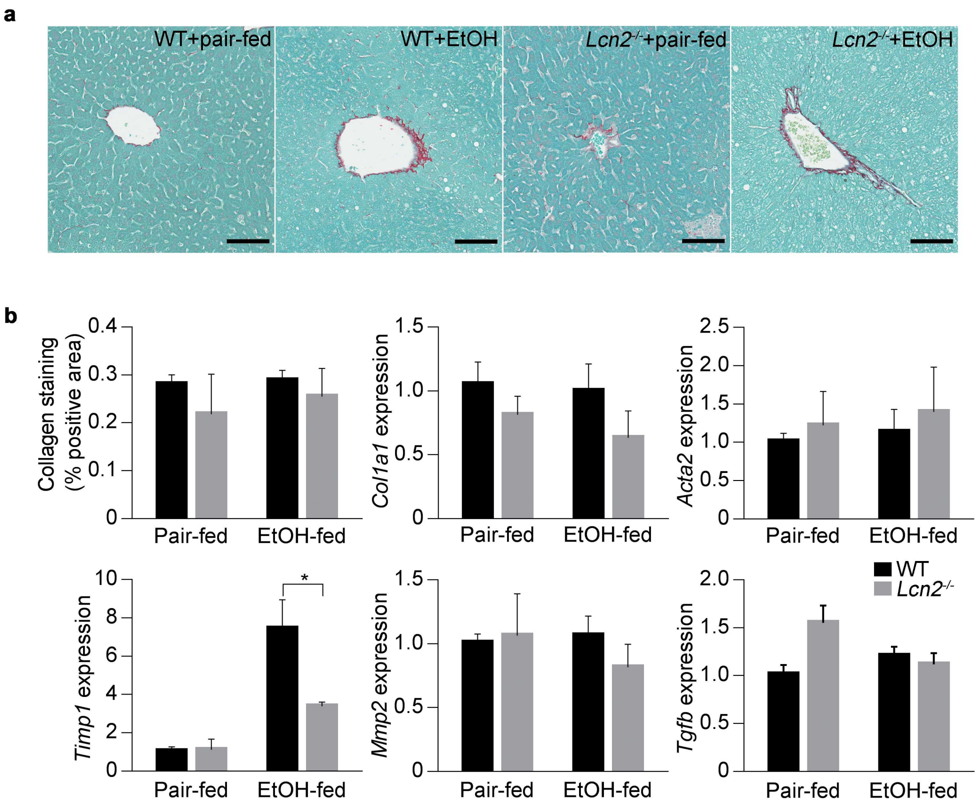

Supplementary figure 4

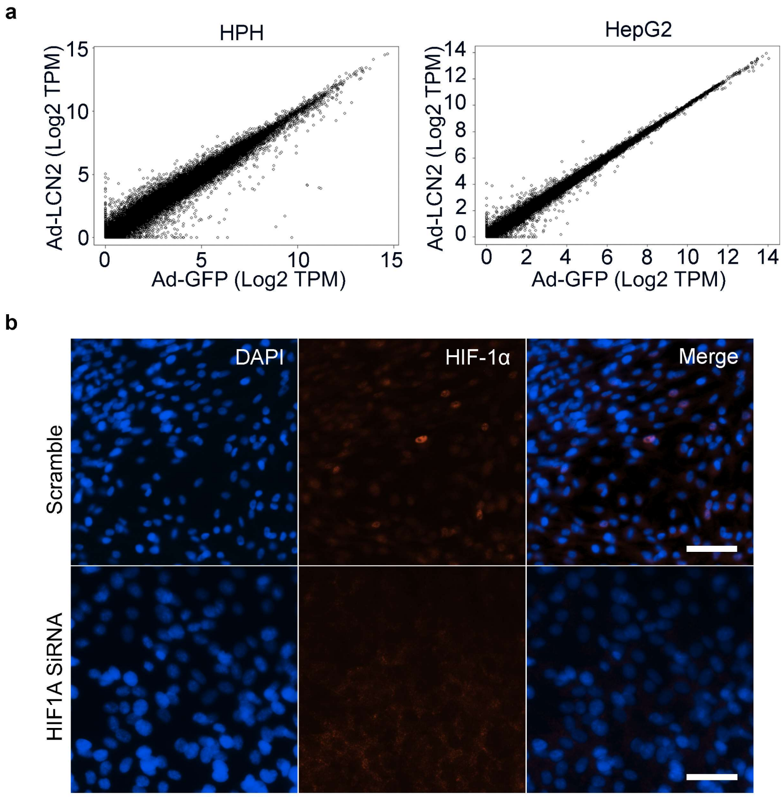

Supplementary figure 5

a

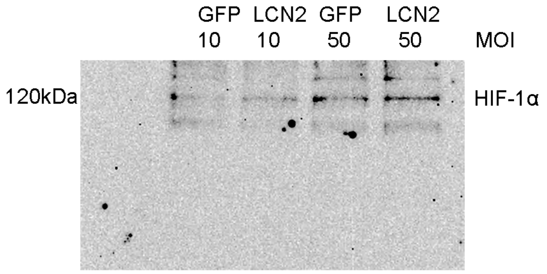

b

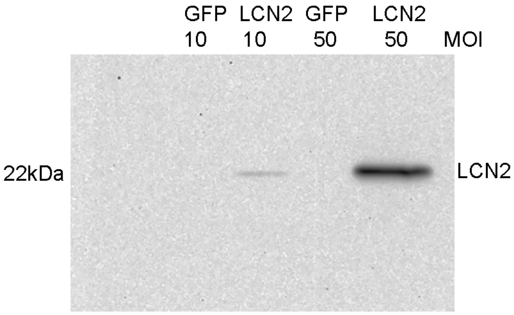

C

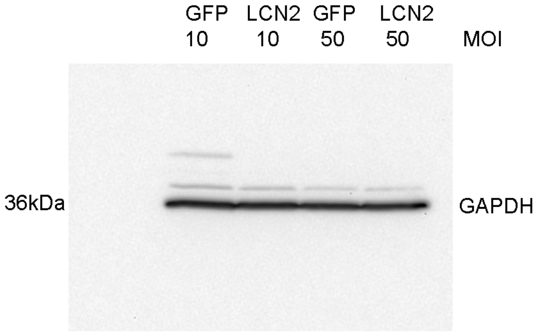

Supplement: Supplementary file 1 — Supplementary Information [file 41598_2020_72172_MOESM1_ESM.pdf]
